# A New Subpopulation of Extracellular Vesicles Harvested from Osteogenically Induced Mesenchymal Stromal Cells of Surgical Site-Released Tissue

**DOI:** 10.3390/biom16020289

**Published:** 2026-02-12

**Authors:** Laura-Marie Joly, Tobias Tertel, Andrea Sowislok, Bernd Giebel, Marcus Jäger

**Affiliations:** 1 Chair of Orthopedics and Trauma Surgery, University of Duisburg-Essen, 45147 Essen, Germany; 2 Institute for Transfusion Medicine, University Hospital Essen, 45147 Essen, Germany; 3 Department of Orthopaedics, Trauma and Reconstructive Surgery, Marien Hospital Mülheim a. d Ruhr, 45468 Mülheim a.d. Ruhr, Germany; 4 Department of Orthopaedics, Trauma and Reconstructive Surgery, Philippus Stift, 45147 Essen, Germany

**Keywords:** surgical site released tissue, bone regeneration, extracellular vesicles, vacuum suction

## Abstract

Impaired bone healing is a major challenge in orthopedic and trauma surgery, often causing long-term disability and high costs. While autologous bone grafting is the gold standard, it is limited by donor site morbidity, low availability, and surgical risks. As an alternative, surgical site-released tissue (SSRT) collected intraoperatively offers a readily available source of regenerative cells and bioactive factors. This study investigates the potential of SSRT-derived mesenchymal stromal cell (MSC)-like cells and their extracellular vesicles (EVs) to support bone healing in a cell-free approach. SSRT samples from 30 patients undergoing elective hip replacement were collected using a surgical vacuum filter. MSC-like cells were isolated and characterized based on International Society for Cellular Therapy (ISCT) criteria. Interestingly, many SSRT-derived MSC-like cells expressed CD34, a marker typically absent in cultured MSCs but linked to tissue-resident stromal cells, suggesting distinct regenerative properties. These cells also showed slow proliferation rates (P1: 8.7 ± 3.2 days; P2: 8.2 ± 5.4 days). EVs were isolated from osteogenically stimulated (EVs^MSC/O+^) and unstimulated (EVs^MSC/O−^) MSCs over three weeks. Antibody profiling revealed distinct cargo compositions, with a notable enrichment of CD13+ EVs in the stimulated group. Further in vivo and functional studies are needed to clarify underlying mechanisms and confirm therapeutic efficacy.

## 1. Introduction

Critical-size bone defects or delayed bone healing based on trauma, infection, or tumor may result in pseudarthrosis and thus lead to disability in affected individuals. The treatment options are limited due to the lack of osteoinductive properties of bone substitutes or allografts. In contrast, autologous bone grafting has osteoinductive properties but is limited in resources and associated with relevant comorbidity of the patient. In addition, sufficient bone regeneration is also required to fix cementless endoprosthesis. Here, delayed osteointegration may result in relevant micromovements and final implant failure [1]. Patients with diminished bone regeneration and repair capacities due to age, systemic diseases, or other conditions are especially at risk for insufficient bone healing [2]. Furthermore, critical-size bone defects are frequently observed in any age. Avascular osteonecrosis and iatrogenic bone defects complete the broad spectrum of hard-to-treat local bone loss. Thus, there is a strong need for bone regenerative strategies which are promising for clinical use. Here, the application of mesenchymal stromal cells (MSCs), the re-transplantation of autologous surgical site-released tissue (SSRT) and its components, and the use of bone substitutes has gained prominence during the past decades [3,4,5,6,7]. MSCs are known for their high self-renewal capability and multipotency, allowing them to differentiate into osteoblastic, chondrogenic and adipogenic cell types [8]. Independent of whether MSCs originate from bone marrow, adipose tissue, umbilical cord blood, or other tissues, they typically can modulate immune responses systemically as well as at injury sites [9,10,11,12,13]. Initially considered to act via direct intercellular interactions, it turned out that in most settings MSCs exert their therapeutic functions in a paracrine manner, via small extracellular vesicles (sEVs) [14,15]. sEVs, including exosomes and small microvesicles, are membrane-surrounded particles with diameters typically ranging between 70 and 150 nm. At least a proportion of them play key roles in intercellular communication and tissue microenvironment modulation [16,17,18,19]. MSC-EVs, mirroring the regenerative properties of their parental MSCs [20], have emerged as a broadly investigated, cell-free stem cell products providing promising therapeutic effects in many preclinical models including bone formation [21]. The precise mechanisms through which MSC-EVs influence osteogenesis are poorly understood and under investigation.

While EV research dates back to the early 1980s [22,23], limitations in techniques such as flow cytometry, which struggles to detect objects smaller than 200–300 nm, prevented comprehensive analyses of EVs [24]. However, recent technical advancements have enabled detailed studies of various EV subpopulations in different biological samples, including plasma [25]. Techniques such as imaging flow cytometry (IFCM) that can effectively detect EVs labeled with fluorescence-conjugated antibodies have become instrumental in identifying specific sEV subsets, offering new insights into the complex world of EVs [26,27].

Building on this advanced understanding of MSC-EVs, our study explores impacts of induced osteogenesis on the EV release [28,29,30]. In addition, we examined the diversity of MSC-EV subpopulations under osteogenic conditions through single EV analysis, focusing on the expression of relevant surface markers.

## 2. Materials and Methods

### 2.1. Patients

In a prospective non-blinded study, MSCs from SSRT were isolated from patients qualified for primary total hip replacement. The study was approved by the institutional ethical board committee (#22-10899-BO, Medical Faculty, University of Duisburg-Essen, Germany). Prior to surgery, all patients gave their written informed consent.

Following the inclusion criteria, the patient cohort consisted of 30 patients (12 males, 18 females) with advanced osteoarthritis qualified for elective total hip replacement. The mean patient age at the time of surgery was 71 ± 12 years (range: 37–86). Table 1 documents the comorbidity profile of the cohort.

### 2.2. Tissue Collection

**Figure 1:**
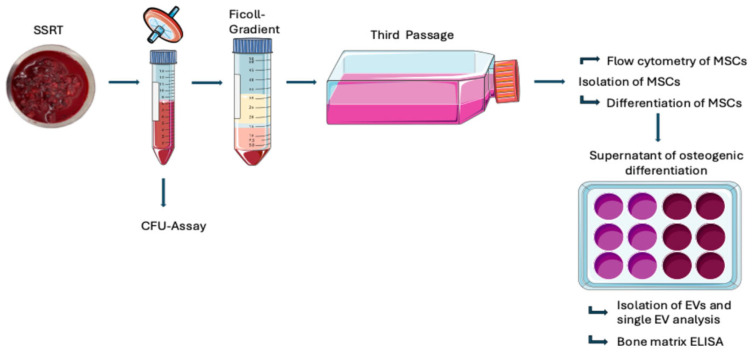
Overview of tissue and cell processing after harvesting of SSRT.

During total hip replacement, using an antero-lateral approach according to Hardinge-Bauer [31,32], locally released tissue (SSRT) was harvested after 20 min by a commercial surgical vacuum suction handle (OP-Flex^TM^FilterFlow^TM^, ConvaTec, Deeside, UK) following femoral neck osteotomy and reaming of the acetabulum. The harvesting device was stored under sterile conditions at 4 °C and transported to the laboratory for further cell isolation and analysis. Figure 1 gives an overview of the protocol.

### 2.3. Generation of Mesenchymal Stromal Cells (MSC)

The aforementioned tissue was processed under sterile conditions within 1 h after harvesting. The MSC generation procedure includes initial removal of fibrin clots and tissue fragments adhered to the surface of the harvesting device, weighing, and incubation with streptokinase (Sigma-Aldrich, Deisenhofen, Germany) diluted with phosphate-buffered saline (PBS; 300 Units/10 mL PBS; Thermo Fisher Scientific, Waltham, MA, USA). Next, mononuclear cells were isolated following Ficoll density gradient centrifugation (Ficoll Plaque^TM^ Plus, density 1.077 g/mL, GE Healthcare, Freiburg, Germany) according to previous studies [4,6,7,33]. Mononuclear cells were cultivated in T25 tissue flasks in a low-glucose DMEM (Gibco, Thermo Fisher Scientific) cell culture medium containing 10% fetal calf serum (FCS; Biochrome, Berlin, Germany) supplemented with 1% penicillin-streptomycin (Sigma-Aldrich). Medium exchange intervals were 3 and 4 days (twice a week). For the identification of MSCs, we complied with the minimal criteria set by the International Society for Cellular Therapy (ISCT), which include plastic adherence, specific surface marker expression, and trilineage differentiation capabilities, thereby ensuring accurate MSC characterization [34].

### 2.4. Self-Renewal (Colony-Forming Unit (CFU) Assay)

To evaluate the colony formation potential of cells derived from surgically released soft tissue (SSRT), CFU assays were performed using freshly isolated cells. Following PBS washing and streptokinase treatment, either 1 × 10^6^, 4 × 10^6^, and 10 × 10^6^; or 10 × 10^6^, 15 × 10^6^, and 20 × 10^6^ cells were seeded per well in standard 6-well culture plates, each condition set up in duplicate. The medium was changed every 3rd day and on day 14. For the analysis, cells were washed with PBS and labeled by incubation in 0.5% crystal violet (Serva, Heidelberg, Germany) in 20% methanol for 30 min at RT followed by rinsing with distilled water. Colonies were defined as circular arrangements of more than 50 stained cells.

### 2.5. Cellular Proliferation (Generation Time)

After Ficoll density gradient centrifugation, MNCs were seeded with a density of 8.6 ± 6.3 × 10^6^ cells/cm^2^ in a T25 flask (passage 0) and cultivated at 37 °C in 5% CO_2_. After reaching a confluency of 80%, the cells were detached and counted (passage 1). The cells were then split and seeded again, this time in a T75 flask. The cellular doubling time was determined for passage 1 and passage 2.

### 2.6. Flow Cytometry of MSCs

After a cultivation period of 4 weeks (3rd passage), cells were dissociated by enzymatic digestion using Accutase (600 U/mL, Gibco, Thermo Fischer, Dreieich, Germany) from the flasks, centrifuged at 300× *g* for 5 min at RT, and suspended in 50 μL PBS containing 3% FCS. After counting the cells, aliquots of 1 × 10^6^ cells were incubated with antibodies against HLA-DR, CD14, CD31, CD34, CD44, CD73, CD90 and CD105 for 30 min on ice without being exposed to light. Isotype controls were used to determine nonspecific signals. The analysis was performed using a CytoFLEX S cytometer (Software CytExpert 2.3; Beckman Coulter, Krefeld, Germany). Obtained data was analyzed with Kaluza Analysis 2.1 software (Beckman Coulter). Detailed information on the antibodies used are provided in Supplementary Materials, Table S1.

### 2.7. Differentiation of MSCs

Osteogenic, chondrogenic and adipogenic differentiation capabilities were identified for each obtained MSC preparation by applying typical in vitro stimulation protocols [6] with the respective media followed by representative cytochemical staining. Unstimulated cells served as controls in all groups.

Osteogenic stimulation: cells were seeded at a density of 2 × 10^5^ cells per well in a 12-well plate. Upon reaching 80% confluence the culture medium was replaced with an osteogenic differentiation medium (Thermo Fisher Scientific; #A10072-01). After a period of 3 weeks, Alizarin Red staining (Lifeline Cell Technology, Frederick, MD, USA; #CM-0058) was performed.

Chondrogenic stimulation: A high-density micro mass culture of (1.6 × 10^7^ viable cells/mL) was prepared. Afterwards, the cells were seeded at a density of 8 × 10^5^ cells per well in a 96-well plate and a chondrogenic induction medium (Thermo Fisher Scientific; #A10071-01) was added. After 3 weeks, the cells were stained using Alcian Blue (Roth, Karlsruhe, Germany; #3082.2).

Adipogenic stimulation: cells were seeded at a density of 4 × 10^5^ cells per well in a 12-well plate. Once the cells reached 80%, an adipogenic induction medium (Thermo Fisher Scientific; #A10070-01) was added. After 3 weeks, the cells were stained with Oil Red-O (Sigma-Aldrich; #O0625).

### 2.8. Preparation of Extracellular Vesicles (EV)

Small extracellular vesicles (sEVs) were prepared from the cell culture supernatants of osteogenically induced MSCs (harvested at P3, as seen in Figure 1) and unstimulated controls that were collected over a period of 3 weeks. Supernatant underwent centrifugation for 5 min at 900× *g*, followed by 15 min at 2000× *g* at 4 °C. Afterwards, it was filtered through a 0.22 µm filter, transferred into ultracentrifuge (UC) tubes, topped up with 0.9% NaCl, and centrifuged for 2 h and 10 min at 100,000× *g*. The obtained EV pellets were resuspended in 50 µL 10 mM HEPES in 0.9% NaCl buffer.

### 2.9. Single EV Analysis by Imaging Flow Cytometry

Imaging flow cytometry (IFCM) analyses for sEVs were conducted for each preparation. The analyses were performed using an Amnis ImageStreamX Mark II Flow Cytometer (AMNIS/Luminex, Seattle, WA, USA). The MSC-EV preparations were stained as described previously [27]. An extended antibody panel was used to identify different EV subtypes. Specifically, antibodies against CD41, PS, CD61, CD59, CD81, CD105, CD9, CD24, CD13, CD100, Annexin A2, CTLA-4, PD-L1, CD53, CD171, CD29, CD63, CD90, CD44, CD235a, CD31, CD200, HLA-ABC and HLA-DR. Briefly, for each staining, 2.5 µL of the MSC-EV preparations were stained with 10 µL of an antibody solution (antibodies diluted in PBS), targeting specific EV markers, and incubated for 1 h at RT in the dark. EV analyses were conducted in accordance with the MIFlowCyt-EV recommendations [18]. To confirm antibody specificity and exclude artifacts, detergent lysis controls and buffer-only samples (without EVs) were included. Unstained samples and buffer controls without MSC-EVs served as additional controls. Stained samples were diluted appropriately with PBS before analysis. For each preparation, 8 different staining procedures were performed. Details of the staining procedure and antibodies used are provided in the Supplementary Materials Tables (Supplementary Materials, Table S2). Due to limitations in sample volumes, samples from 15 patients were analyzed (*n* = 15).

For IFCM analysis, samples were applied to U-bottom 96-well Falcon plates (Corning GmbH, Kaiserslautern, Germany) and analyzed in triplicate. Each well was subjected to a 5 min acquisition time at 60× magnification, with a low flow rate and the “Remove Beads” option deactivated. Data were acquired using IDEAS 6.2 software (Cytek Bioscience, Fremont, CA, USA). Fluorescence intensities and side scatter values were plotted, and the spot counting feature was employed for swarm detection analysis. Multiplets were excluded, and events with low side scatter (<500) and fluorescence intensity higher than 300 were considered for concentration calculations [27].

### 2.10. Statistical Analysis

Statistical analysis was conducted using Graph Pad Prism v10 (GraphPad Prism Software, San Diego, CA, USA). Continuous variables such as patients’ age, sample weight, MNC number and generation time are presented as mean ± standard deviation, and categorical variables such as gender are presented as frequency and percentage. Repeated measures data were analyzed using a mixed-effects model when values were missing, or a two-way ANOVA when there were no missing values, followed by Šidák multiple comparison testing. The Kruskal–Wallis test was used to detect changes in the kinetics over the course of three weeks. Differences were considered significant at *p* < 0.05. By utilizing these statistical measures and presentation formats, the study seeks to provide a comprehensive and accurate description of the data, facilitating the interpretation and communication of the findings.

## 3. Results

### 3.1. SSRT Samples Can Serve as Cell Source for MSC-like Cells

The average weight of the surgical site-released tissue (SSRT) from the 30 samples was 15 ± 6.7 g. Cells harvested from the tissues yielded a mean of 4.8 ± 2.6 × 10^10^ cells per sample, corresponding to 3.7 ± 2.8 × 10^9^ cells per gram of tissue. A colony-forming unit (CFU) assay performed prior to Ficoll-gradient centrifugation revealed colony formation capacity, with an average of 0.5 ± 0.4 ×10^6^ CFU per sample. Based on these data, the theoretical number of potential MSCs in the samples was calculated, resulting in an average number of 3.3 ± 4.7‰ cells per sample or 0.4 ± 0.9‰/g of tissue.

Following Ficoll-gradient centrifugation, on average, 2.3 ± 1.5 × 10^8^ mononuclear cells corresponding to 1.5 ± 0.9 × 10^7^ cells per gram of the SSRT samples were obtained. Upon culturing mononuclear cells under MSC-generation conditions, MSC-like cells were obtained in all cases. The population doubling time of obtained MSC-like cells were 8.7 ± 3.2 days in P1 and 8.2 ± 5.4 days in P2. The results are summarized in Table 2.

### 3.2. SSRT-Derived Cells Are Mixed Stromal Cultures

**Figure 2:**
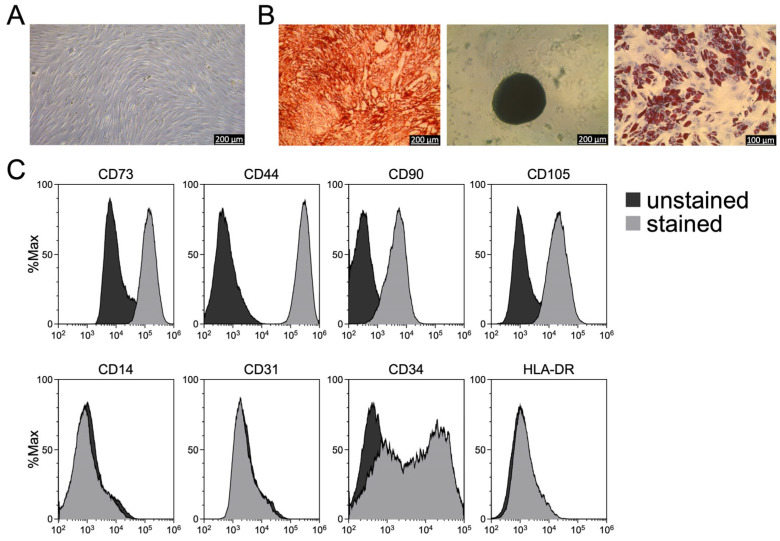
Phenotypic characterization and analysis of the differentiation potential demonstrate the MSC nature of the expanded cells. (**A**) Morphologies of expanded cells; scale bar = 200 μm. (**B**) From left to right: cells following osteogenic induction and Alizarin Red S staining (scale bar = 200 μm), following chondrogenic induction and Alcian Blue staining (scale bar = 200 μm), and adipogenic-induction and Oil Red O staining (scale bar = 100 μm). (**C**) Flow cytometric analysis of specific cell surface antigens: black, isotype controls; gray, antibody-labeled cells. Images are representative of a series of 15 distinct samples. Percentages indicate the fraction of cells exceeding the fluorescence threshold of the unstained control. Note that for markers with overlapping histograms (e.g., CD90, CD105), the values represent a conservative estimate despite a complete population shift.

To evaluate the expression of cell surface antigens on the MSCs obtained from SSRT samples, flow cytometry was performed using the ISCT-recommended antibody panel for MSC characterization. This included the antibody targeting the positive markers CD73, CD44, CD90, and CD105, as well as the negative markers CD14, CD31, CD34 and HLA-DR. As expected, the expanded cells consistently expressed all positive MSC markers while lacking expression of CD14, CD31 and HLA-DR. Notably, however, a subpopulation of the cells showed detectable expression of CD34, a marker typically absent from bona fide MSCs (Figure 2C).

### 3.3. SSRT-Derived Cells Exhibit Tri-Lineage Differentiation Potential

To determine whether the expanded cells retain characteristic MSC functions despite the presence of CD34 expression in a subpopulation, their differentiation potential along the osteogenic, adipogenic, and chondrogenic lineages was assessed (Figure 2B). In all samples, successful differentiation into cell types representative of each lineage was observed, confirming the multipotent capacity typical of MSCs. These findings indicate that, despite partial CD34 expression given the mixed cell population, the expanded cells fulfill the functional criteria defining bona fide MSCs.

### 3.4. Extracellular Vesicle (EV) Analysis Shows Different Subpopulations of MSC-EVs Depending on Osteogenic Stimulation

We next characterized extracellular vesicles (EVs) and their subpopulations to better understand MSC-mediated communication during bone formation. Imaging flow cytometry identified 15 EV surface markers: HLA-ABC, PS, CD13, CD44, CD81, CD63, CTLA-4, CD24, CD53, CD90, CD200, CD29, CD59, CD100, PD-L1.

#### 3.4.1. Differential EV Marker Expression During Cell Culture of MSC/O+

**Figure 3:**
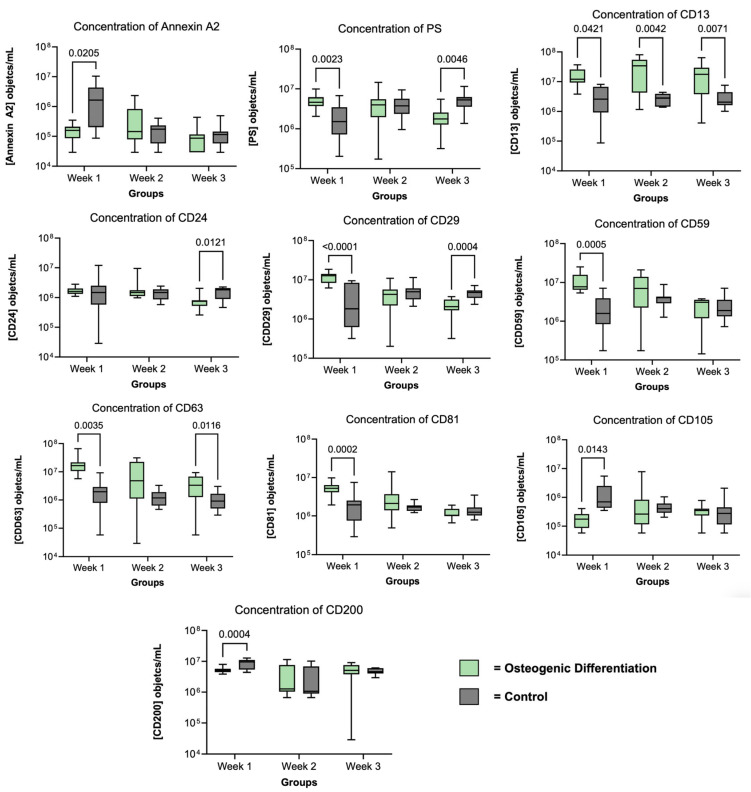
Temporal profile of extracellular vesicle (EV) surface marker concentrations in osteogenically stimulated (MSC/O^+^) and unstimulated control (MSC/O^−^) groups over three weeks. Box plots show median, interquartile range, and minimum/maximum values for each marker (objects/mL of cell culture supernatant). Significant differences between groups were determined using a mixed-effects model followed by Šidák correction (*n* = 15).

Within the first week of cell culture, significant differences between stimulated and unstimulated MSCs can be detected. While the concentration of CD81, CD63, CD59, CD29 and PS is high for the osteogenically stimulated samples and low for the unstimulated control group, CD200, CD105 and Annexin A2 show the opposite distribution. Here, the osteogenically stimulated group shows lower concentrations compared to the unstimulated control group. In the third week of cell culture, however, there are significantly higher concentrations of CD63 and CD13 in the EVs compared to osteogenically stimulated MSCs, while the concentrations of CD29, PS and CD24 are statistically significantly higher in the unstimulated control. Figure 3 provides an overview of these early differences between stimulated and unstimulated MSCs and illustrates the temporal evolution of EV marker expression across three time points (weeks 1–3). The figure thus offers a global view of EV marker dynamics under osteogenic induction, while detailed week 1 → week 3 kinetics for each group are summarized in Table 3 and Table 4. Supplementary Materials Table S3 contains the complete statistical comparisons.

#### 3.4.2. EV Kinetics over Three Weeks

While Figure 3 shows the time-dependent overall distribution of EV surface markers in both conditions, Table 3 and Table 4 summarize the specific kinetic changes between week 1 and week 3 for the osteogenically stimulated (MSC/O^+^) and control (MSC/O^−^) groups, respectively.

**Figure 4:**
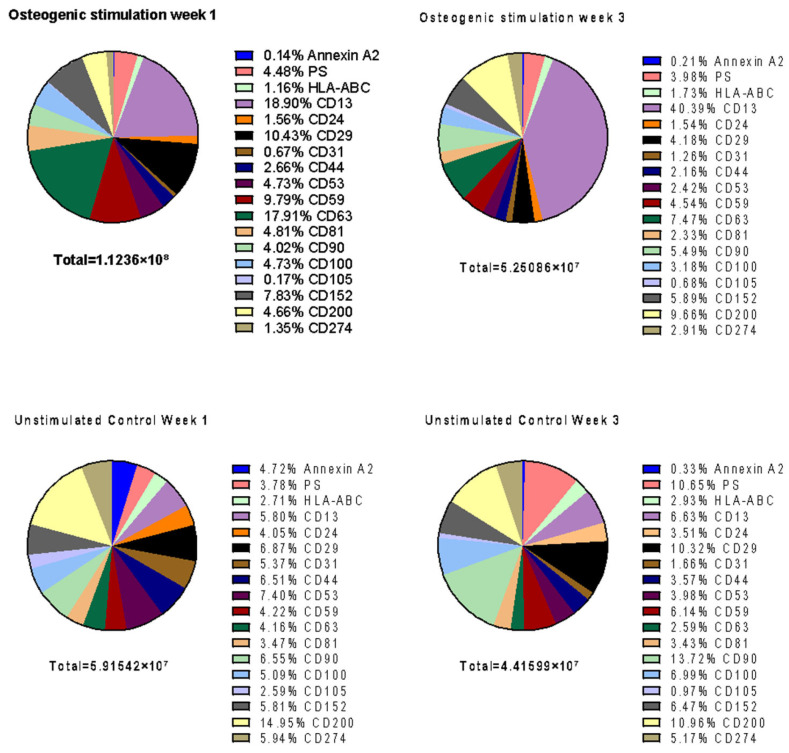
The figure summarizes the composition of EV^MSC/O+^ and EV^MSC/O−^ after week 1 and 3. The pie charts show relative concentrations [%] of defined surface markers.

When comparing the concentrations between week one and week three, there are statistically significant differences for PS^+^ (*p* < 0.01), CD59^+^ (*p* < 0.001), CD81^+^ (*p* < 0.0001), CD24^+^ (*p* < 0.001), CD100^+^ (*p* < 0.01), CD53^+^ (*p* < 0.0001), CD29^+^ (*p* < 0.0001), CD63^+^ (*p* < 0.01), CD44^+^ (*p* < 0.01), CD105^+^ (*p* < 0.05), and CD200^+^ (*p* < 0.01) objects. More detailed information is provided in Figure 4.

While the concentration of PS, CD24, CD29, CD44, CD53, CD59, CD63, CD81 and CD100 in osteogenically stimulated MSC decreased in EV^MSC/O+^ over 3 weeks, the unstimulated control (EV^MSC/O−^) shows a corresponding decrease in Annexin A2, CD53, CD105, and CD200. CD53 was the only marker in which both EV^MSC/O+^ and EV^MSC/O−^ show the same kinetic. The concentrations of the other surface markers behave in an antiparallel manner. This leads to a relevant change in EV composition between EV^MSC/O+^ and EV^MSC/O−^ with a significantly higher percentage of CD13 in EV^MSC/O+^ at week 3 (Figure 4). The results suggest differential EV expression in osteogenically induced mesenchymal stromal cells. The MSC-EVs of the osteogenically stimulated group exhibit a fundamentally altered composition of surface antigens. This suggests a different subgroup of MSC-EVs with potentially osteogenic properties.

## 4. Discussion

Our study investigated extracellular vesicles (EVs) derived from mesenchymal stromal cells (MSCs) isolated from surgical site-released tissue (SSRT). The robust cell yield and high proliferation capacity underline the feasibility of using autologous MSCs and their EVs in clinical bone regeneration. The significant baseline cell counts we observed support the practicality of harvesting MSCs from surgical sites, consistent with earlier reports demonstrating their multipotency and regenerative potential [4,5,6,7,33].

### 4.1. SSRT as Inhomogeneous Sample for Harvesting MSCs

An unexpected observation in our study was the substantial presence of CD34+ cells among the MSC-like cells isolated from SSRT. Traditionally, CD34 has been considered a negative marker for MSCs, mainly associated with hematopoietic and endothelial progenitors and it is entirely possible that SSRT-derived cells contribute a mixed stromal cell population including those cell types. However, emerging evidence challenges this rigid classification. Recent studies have shown that tissue-resident MSCs, particularly those in their native microenvironments, can express CD34, and that this expression is often lost upon extended in vitro expansion [35,36]. Our finding of CD34 positivity suggests that SSRT-derived MSCs may represent a more primitive or less culture-adapted MSC population, potentially retaining functional properties that are lost in long-term cultured MSCs. Interestingly, Lin et al. argued that CD34 expression does not negatively impact MSC functionality and may even reflect an enhanced regenerative potential [37]. Moreover, the coexistence of CD34+ cells in our cultures aligns with data suggesting that CD34 expression might be more related to a native, undisturbed MSC phenotype rather than being purely a hematopoietic marker [35,36,38] This is underlined by the possibility of all MSC samples to differentiate into osteogenic, chondrogenic, and adipogenic lineages, confirming their functional plasticity and supporting their therapeutic potential in regenerative applications [39].

This observation has significant implications, as the potential supportive or synergistic roles of mixed stromal cell populations could enhance tissue regeneration and bone healing outcomes, adding another layer of complexity and therapeutic promise to SSRT-derived MSC applications. It also implies that the isolation of a specific cell type may not be necessary to utilize the therapeutic aspects of exosomes.

### 4.2. Osteogenically Induced MSC Release a Different Subpopulation of EVs

Dynamic shifts in EV surface marker profiles during osteogenic induction highlight the strong influence of cellular stimulation on EV cargo and potential functionality. The observed stable upregulation of CD13 in EV^MSC/O+^ further emphasizes its possible role in osteogenesis and tissue repair [37,40]. Other markers, such as PS and CD24, showed differential kinetics over three weeks, highlighting dynamic changes in EV subpopulations that may reflect functional specialization toward bone formation or modulation of the microenvironment. Markers such as Annexin A2, PS, CD13, CD24, CD29, CD59, CD63, CD81, CD105, and CD200 have established roles in osteoclast formation, osteoblast differentiation, cell adhesion, or signaling, reinforcing the potential of MSC-derived EVs as modulators of bone regeneration. The combination of SSRT-derived MSCs and their EVs therefore represents a promising approach for autologous, cell-free bone regeneration. EVs carrying osteogenesis-associated markers may enhance mineralized matrix formation while potentially reducing the risks and logistical challenges associated with direct cell transplantation.

### 4.3. Limitations and Future Aspects

While our study provides a preliminary assessment of the regenerative potential of SSRT-derived MSC-EVs based on marker profiles, their functional potential remains to be fully validated. A limiting factor in our study is the advanced osteoarthritis of all patient samples used—a control with patients undergoing a total hip replacement due to a hip fracture could be a valuable control in upcoming studies. It is nevertheless important to assess the potential in cells of the patients that are most likely affected and could potentially profit from such therapies in the near future. Another important aspect is the possible donor-to-donor variability due to the complex origin tissue and the resulting problem in reproducibility and batch consistency. Further studies with a higher number of samples with comparison in pooling are necessary.

Future work could explore the effects of EVs on osteogenic differentiation and proliferation in target cells, assess their contribution to bone healing in vivo using animal models, and investigate which surgical procedures yield MSCs and EVs suitable for clinical application. Mechanistic studies on specific EV markers, such as CD13, PS, and CD24, would further clarify how these vesicles modulate osteogenesis, osteoclast regulation, and paracrine signaling within the bone microenvironment. Furthermore, the verification of the functional aspects of the regenerative potential of EVs would strengthen the evidence of future studies.

## 5. Conclusions

In summary, our study underscores the potential of SSRT-derived MSCs and their EVs as promising candidates for bone regeneration. EVs mediated from osteogenically induced MSCs lead to a dynamic shift in EV surface marker profiles reflecting functional specialization and supporting their therapeutic potential. Notably, the unexpected and robust expression of CD34 among MSCs suggests that these cells might represent a more native, highly regenerative stromal population. Exosome-mediated osteogenesis remains an important topic to be further investigated. These findings advocate for revisiting the MSC definition and support further exploration of SSRT-derived MSCs and their EVs in preclinical and clinical bone healing models.

## Figures and Tables

**Table 1:** Patient characteristics and comorbidities.

Parameter	Number (%)
Patients	30
Age (years)	71 ± 12
Gender	
Female	18 (60%)
Male	12 (40%)
Comorbidities	
cardiovascular diseases	16 (48%)
diabetes mellitus	2 (6%)
obesity and hyperlipidaemia	9 (27%)
pulmonary diseases	4 (12%)
tumor in medical history (no active neoplasm)	3 (9%)
chronic anemia	4 (12%)
renal insufficiency (chronic and acute)	4 (12%)

**Table 2:** Characteristics of SSRT samples and derived cells (mean ± SD). —: not available.

Parameter	Value per Sample	Value per g of Tissue
SSRT weight	15 ± 6.7 g	—
Isolated cells	4.8 ± 2.6 × 10^10^	3.7 ± 2.8 × 10^9^
Colony-forming cells (CFU)	3.3 ± 4.7‰	0.4 ± 0.9‰
Mononuclear cells	2.3 ± 1.5 × 10^8^	1.5 ± 0.9 × 10^7^
Doubling time P1	8.7 ± 3.2 d	—
Doubling time P2	8.2 ± 5.4 d	—

**Table 3:** Kinetic changes in EV surface marker concentrations in osteogenically stimulated MSCs (MSC/O^+^) between week 1 and week 3. Data are presented as mean ± SD (objects/mL). Arrows indicate direction of change (decrease) over time. All markers show significantly decreased concentrations at week 3 compared with week 1 (mixed-effects model, Šidák post hoc test).

Antibody	Week 1	Week 3		*p*-Value
PS	5.03 × 10^6^ ± 2.12 × 10^6^	2.09 × 10^6^ ± 1.37 × 10^6^	↓	0.0005
CD24	1.76 × 10^6^ ± 5.25 × 10^5^	8.07 × 10^5^ ± 4.78 × 10^5^	↓	0.0001
CD29	1.17 × 10^7^ ± 3.45 × 10^6^	2.20 × 10^6^ ± 1.04 × 10^6^	↓	<0.0001
CD44	2.99 × 10^6^ ± 1.45 × 10^6^	1.13 × 10^6^ ± 8.09 × 10^5^	↓	0.0022
CD53	5.31 × 10^6^ ± 1.20 × 10^6^	1.27 × 10^6^ ± 7.61 × 10^5^	↓	<0.0001
CD59	1.01 × 10^7^ ± 6.56 × 10^6^	2.38 × 10^6^ ± 1.31 × 10^6^	↓	<0.0001
CD63	2.01 × 10^7^ ± 1.69 × 10^7^	3.92 × 10^6^ ± 3.07 × 10^6^	↓	<0.0001
CD81	5.40 × 10^6^ ± 1.99 × 10^6^	1.22 × 10^6^ ± 3.44 × 10^5^	↓	<0.0001
CD100	5.31 × 10^6^ ± 3.09 × 10^6^	1.67 × 10^6^ ± 4.63 × 10^5^	↓	<0.0001

**Table 4:** Kinetic changes in EV surface marker concentrations in unstimulated MSCs (MSC/O^−^) between week 1 and week 3. Data are presented as mean ± SD (objects/mL). Arrows indicate direction of change (↑ increase, ↓ decrease). Most markers show a decrease over time, with PS being the only one showing an increase. Statistical analysis as in Table 3.

Antibody	Week 1	Week 3		*p*-Value
annexin A2	2.79 × 10^6^ ± 3.22 × 10^6^	1.44 × 10^5^ ± 1.27 × 10^5^	↓	0.0018
PS	2.24 × 10^6^ ± 1.93 × 10^6^	4.70 × 10^6^ ± 2.99 × 10^6^	↑	0.0248
CD53	4.38 × 10^6^ ± 2.90 × 10^6^	1.76 × 10^6^ ± 1.35 × 10^6^	↓	0.0045
CD105	1.53 × 10^6^ ± 1.55 × 10^6^	4.27 × 10^5^ ± 5.48 × 10^5^	↓	0.0017
CD200	8.84 × 10^6^ ± 2.71 × 10^6^	4.84 × 10^6^ ± 1.05 × 10^6^	↓	0.0131

## Data Availability

Data are contained within the article or Supplementary Material.

## Supplementary Materials

The following supporting information can be downloaded at: https://www.mdpi.com/article/10.3390/biom16020289/s1, Figure S1: Sample harvesting in the operating field and sterile sample preparation; Figure S2: Osteogenically stimulated MSC-EVs regarding their kinetic over the course of three weeks in comparison to an unstimulated control group; Table S1: List of monoclonal antibodies against defined surface markers used in the flow cytometry analysis of MSCs; Table S2: List of monoclonal antibodies against defined surface markers used in the EV analysis; Table S3: EV-associated markers investigated in osteogenically stimulated MSC-EVs and a paired control group compared at week 1; Table S4: EV-associated markers investigated in osteogenically stimulated MSC-EVs and a paired control group compared at week 3.
