# Supplementary material for: A New Subpopulation of Extracellular Vesicles Harvested from Osteogenically Induced Mesenchymal Stromal Cells of Surgical Site-Released Tissue
Source: Biomolecules. 2026 Feb 12;16(2):289. doi: 10.3390/biom16020289 (PMC12938177; doi:10.3390/biom16020289)
Supplement: Supplementary file 1 [file biomolecules-16-00289-s001.zip › biomolecules-4141728-supplementary.pdf]

biomolecules

MDPI

# Supplementary Material

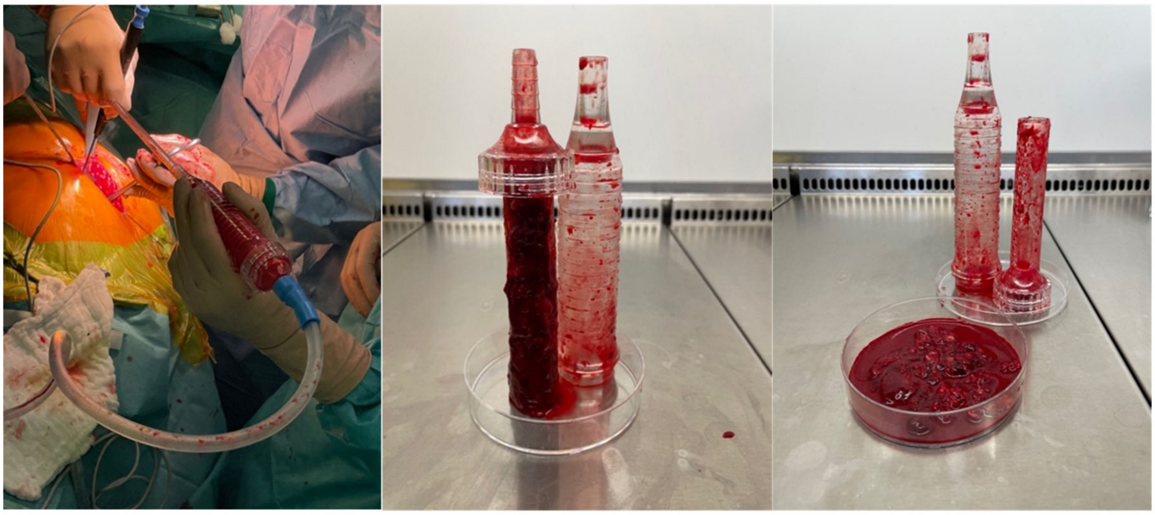

**Figure S1.** Sample harvesting in the operating field and sterile sample preparation.

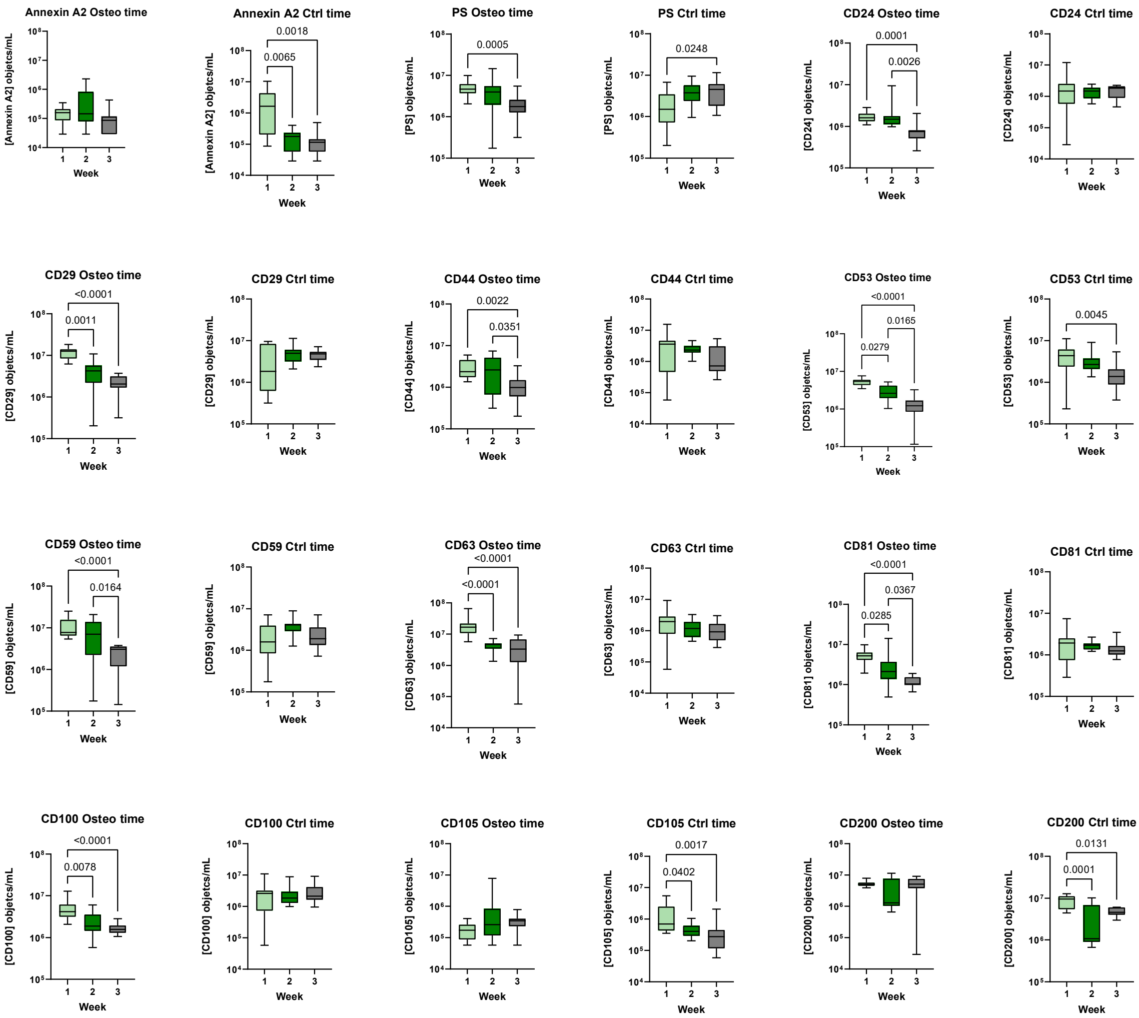

**Figure S2.** Osteogenically stimulated MSC-EVs regarding their kinetic over the course of three weeks in comparison to an unstimulated control group. The concentration is given as objects/mL cell culture supernatant. Data are presented as box plots with median. Significances were determined using a mixed-effects-model, followed by Šidák multiple comparison testing. P<0.05 = *, p<0.01 = **, p<0.001 = ***, p<0.0001 = ****, n=15.

**Table S1.** List of monoclonal antibodies against defined surface markers used in the flow cytometry analysis of MSCs.

| Antibody | Conjugate | Clone | Order Nr. | Company | Isotype |
|---|---|---|---|---|---|
| **CD73** | Fitc | AD2 | 11-0739-42 | Invitrogen | Mouse IgG1 k |
| **CD31** | PE | WM59 | 303106 | BioLegend | Mouse IgG1 k |
| **HLA-DR** | ECD | Immu-357 | IM3636 | Beckmann Coulter | Mouse IgG1 k |
| **7 AAD** | | | A07704 | Beckmann Coulter | |
| **CD44** | APC | G44-26 | 559942 | BD Pharmingen | Mouse IgG2b k |
| **CD34** | APC/Fire 750 | 581 | 343536 | BioLegend | Mouse IgG1 k |
| **CD105** | BV421 | 43A3 | 323219 | BioLegend | Mouse IgG1 k |
| **CD14** | Pacific Orange | MEM-15 | PO-293 | Exbio | Mouse IgG1 |
| **CD90** | BV650 | 5E10 | 328144 | BioLegend | Mouse IgG1 k |
| **CD45** | Alexa Flour 700 | HI30 | 304024 | BioLegend | Mouse IgG1 k |

**Table S2.** List of monoclonal antibodies against defined surface markers used in the EV analysis.

| Antibody | Conjugate | Clone | Order Nr. | Company | Isotyp |
|---|---|---|---|---|---|
| **CD63** | APC | MEM-259 | 1A-343-T100 | Exbio | mouse IgG1 |
| **CD81** | FITC | JS64 | B25329 | Beckman Coulter | mouse IgG2a |
| **CD200** | BV 510 | MRC OX-104 | 563254 | BD Biosciences | Mouse IgG1, κ |
| **HLA-ABC** | FITC | B9.12.1 | IM1838U | Beckman Coulter | IgG2a Mouse |
| **CD13** | PE | QA19A12 | 111003 | BioLegend | rat IgG2a, κ |
| **CD24** | PE | M1/69 | 130-102-732 | Miltenyi Biotec | rat IgG2bκ |
| **CD53** | PE | MEM-53 | 1P-227-T100 | Exbio | Mouse IgG1 |
| **CD100** | PE | 133-1C6 | 1P-772-T100 | Exbio | mouse IgM |
| **PS** | AF 488 | 1H6 | 16-256 | Sigma-Aldrich | mouse IgG |
| **CD152** | PE | BNI3 | 555853 | BD Biosciences | Mouse BALP/c IgG2a, κ |
| **CD274** | PE | 29E.2A3 | 329705 | BioLegend | mouse IgG2b, κ |
| **Annexin A2** | PE | D11G2 | 15161 | Cell Signal | Rabbit IgG |
| **CD29** | APC | REA1060 | 130-118-122 | Miltenyi | human IgG1 |
| **CD59** | FITC | p282(H19) | 555763 | BD Biosciences | Mouse IgG2a, κ |

**Table S3.** EV-associated markers investigated in osteogenically stimulated MSC-EVs and a paired control group compared at **week 1.** Only statistically significant differences shown. Data are provided as mean ± SD.

| Surface Marker | Osteogenically stimulated MSC-EVs Mean ± SD; week 1 | Unstimulated MSC-EVs Mean ± SD; week 1 | p-value |
|---|---|---|---|
| **CD81** | $5.4 \times 10^6 \pm 1.99 \times 10^6$ | $2.05 \times 10^6 \pm 1.9 \times 10^6$ | 0.0002 |

https://doi.org/10.3390/biom16020289

| | | | |
|---|---|---|---|
| **CD63** | 2.01 x $10^7$ ± 1.69 x $10^6$ | 2.46 x $10^6$ ± 2.5 x $10^6$ | 0.0035 |
| **CD59** | 1.1 x $10^7$ ± 6.56 x $10^6$ | 2.5 x $10^6$ ± 2.17 x $10^6$ | 0.0005 |
| **CD29** | 1.17 x $10^7$ ± 3.45 x $10^6$ | 4.06 x $10^6$ ± 3.91 x $10^6$ | < 0.0001 |
| **PS** | 5.03 x $10^6$ ± 2.12 x $10^6$ | 2.24 x $10^6$ ± 1.93 x $10^6$ | 0.0023 |
| **CD200** | 5.24 x $10^6$ ± 1.1 x $10^6$ | 8.84 x $10^6$ ± 2.71 x $10^6$ | 0.0004 |
| **CD105** | 1.95 x $10^5$ ± 1.22 x $10^5$ | 1.53 x $10^6$ ± 1.55 x $10^6$ | 0.0143 |
| **CD13** | 2.12 x $10^7$ ± 2.46 x $10^7$ | 3.43 x $10^6$ ± 2.7 x $10^6$ | 0.0421 |
| **Annexin A2** | 1.59 x $10^5$ ± 8.84 x $10^4$ | 2.79 x $10^6$ ± 3.22 x $10^6$ | 0.0205 |

**Table S4.** EV-associated markers investigated in osteogenically stimulated MSC-EVs and a paired control group compared at **week 3**. Only statistically significant differences shown. Data are provided as mean ± SD.

| Surface Marker | Osteogenically stimulated MSC-EVs Mean ± SD; Week 3 | Unstimulated MSC-EVs Mean ± SD; Week 3 | p-value |
|---|---|---|---|
| **CD63** | 3.92 x $10^6$ ± 3.07 x $10^6$ | 1.14 x $10^6$ ± 8.36 x $10^5$ | 0.0116 |
| **CD29** | 2.2 x $10^6$ ± 1.04 x $10^6$ | 4.56 x $10^6$ ± 1.45 x $10^6$ | 0.0004 |
| **PS** | 2.1 x $10^6$ ± 1.37 x $10^6$ | 5.5 x $10^6$ ± 2.99 x $10^6$ | 0.0046 |
| **CD13** | 2.12 x $10^7$ ± 1.91 x $10^7$ | 2.93 x $10^6$ ± 2.01 x $10^6$ | 0.0071 |
| **CD24** | 8.08 x $10^5$ ± 4.78 x $10^5$ | 1.55 x $10^6$ ± 6.6 x $10^5$ | 0.0121 |

https://doi.org/10.3390/biom16020289
